# Immunological Findings and Clinical Outcomes of Infants With Positive Newborn Screening for Severe Combined Immunodeficiency From a Tertiary Care Center in the U.S.

**DOI:** 10.3389/fimmu.2021.734096

**Published:** 2021-09-03

**Authors:** Vasudha Mantravadi, Jeffrey J. Bednarski, Michelle A. Ritter, Hongjie Gu, Ana L. Kolicheski, Caroline Horner, Megan A. Cooper, Maleewan Kitcharoensakkul

**Affiliations:** ^1^The Division of Allergy and Pulmonary Medicine, Department of Pediatrics, Washington University School of Medicine, St. Louis, MO, United States; ^2^The Division of Hematology and Oncology, Department of Pediatrics, Washington University School of Medicine, St. Louis, MO, United States; ^3^The Division of Pediatric Rheumatology/Immunology, Department of Pediatrics, Washington University School of Medicine, St. Louis, MO, United States; ^4^The Division of Biostatistics, Washington University School of Medicine, St. Louis, MO, United States

**Keywords:** newborn screen (NBS), severe combined immune deficiency (SCID), TREC, lymphopenia, digeorge

## Abstract

The implementation of severe combined immunodeficiency (SCID) newborn screening has played a pivotal role in identifying these patients early in life as well as detecting various milder forms of T cell lymphopenia (TCL). In this study we reviewed the diagnostic and clinical outcomes, and interesting immunology findings of term infants referred to a tertiary care center with abnormal newborn SCID screens over a 6-year period. Key findings included a 33% incidence of non-SCID TCL including infants with novel variants in *FOXN1*, *TBX1*, *MYSM1, POLD1*, and *CD3E*; 57% positivity rate of newborn SCID screening among infants with DiGeorge syndrome; and earlier diagnosis and improved transplant outcomes for SCID in infants diagnosed after compared to before implementation of routine screening. Our study is unique in terms of the extensive laboratory workup of abnormal SCID screens including lymphocyte subsets, measurement of thymic output (TREC and CD4TE), and lymphocyte proliferation to mitogens in nearly all infants. These data allowed us to observe a stronger positive correlation of the absolute CD3 count with CD4RTE than with TREC copies, and a weak positive correlation between CD4RTE and TREC copies. Finally, we did not observe a correlation between risk of TCL and history of prenatal or perinatal complications or low birth weight. Our study demonstrated SCID newborn screening improves disease outcomes, particularly in typical SCID, and allows early detection and discovery of novel variants of certain TCL-associated genetic conditions.

## Introduction

Severe combined immunodeficiency (SCID) comprises a broad group of genetic disorders of the adaptive immune system in which T cell, and sometimes B and NK cell, development and function are impaired. Patients are susceptible to severe opportunistic infections starting in the first months of life and do not survive past 1 year of age without intervention (1). Overall incidence of SCID has been shown to be roughly 1:58,000 based on 11 screening programs in the United States (2).

SCID newborn screening by the T cell receptor excision circle (TREC) assay is a measure of T cell receptor gene rearrangement and, thus, a marker of naïve T cell production by the thymus. In the United States screening was first implemented in Wisconsin in 2008, and in all 50 states by 2018. This has allowed for earlier diagnosis and treatment, typically with hematopoietic stem cell transplant, gene therapy, or enzyme replacement, and has led to improved survival of infants with SCID (2–6). These outcomes are highlighted in a study from the Primary Immune Deficiency Treatment Consortium which found 94% survival among infants who were transplanted by 3.5 months age, *versus* 50% survival among those who had active infection and were transplanted later than 3.5 months age (7).

SCID screening has also led to the detection of various types of non-SCID, or idiopathic, T cell lymphopenia (TCL) of which the clinical implications and outcomes have been less clear (4, 8). Some of the conditions responsible for non-SCID TCL include chromosome abnormalities, cardiac or gastrointestinal anomalies, prematurity, and prenatal factors such as maternal diabetes or use of immunosuppressive medications (1, 2). In cases where no clear syndrome or genetic cause of SCID is identified, leading to a diagnosis of idiopathic TCL, the decision of whether hematopoietic stem cell transplant (HSCT) would be beneficial becomes challenging, and is guided by the specific immunologic findings in the patient and their evolution over time (9). Though some infants have persistent TCL beyond 2 years of age, others are only transiently lymphopenic, as found by one referral center in New York (10).

SCID screening algorithms and TREC cutoff values vary among different programs, with less stringent threshold values generally allowing for increased detection of non-SCID TCL cases (11). By correlating TRECs with T lymphocyte subsets at the population level, immunologists and public health officials can adjust TREC cutoff values on the newborn screen to most efficiently identify those with TCL (5). Although the correlation between TREC levels and T cell counts is generally strong, it is not always consistent (8, 12).

In this study, we characterize the diagnostic and clinical outcomes of 154 infants referred to St. Louis Children’s Hospital Immunology clinic with positive newborn SCID screens from Missouri and Illinois between July 2014 and March 2020. In doing so, we identified several unique gene variants associated with non-SCID TCL that were not previously reported. We also analyzed a broad range of immunological laboratory data for all infants in the study including lymphocyte subpopulations, TREC copies, CD4^+^ T cell recent thymic emigrants (CD4RTE), and naïve CD4^+^ cell percentage. Finally, we assessed the significance of prenatal complications and birth weight in relation to the incidence of TCL.

## Methods

Data was collected on patients referred from Illinois and Missouri. The newborn SCID screening program started in July 2014 in Illinois and in January 2017 in Missouri. Both states utilize an in-house multiplex assay involving the CDC *in situ* method for detection of TREC by RT-PCR with RNaseP as the control. Our institution is one of 3 referral centers in Missouri for positive SCID screens and receives roughly a third of the total statewide referrals. Referrals are made based on patient and primary care provider preference and not on the TREC level or any geographical restrictions. While the proportion of Illinois referrals that we receive is unclear, these patients generally reside in southern Illinois, where our institution is the nearest SCID referral center. Newborn screening data at the state level is organized by specimen rather than by patient so the total number of individuals screened statewide was unavailable. A positive newborn screen for SCID in Illinois was defined as a TREC level of 250 copies/μL or less, and in Missouri as a cycle threshold value of 37 or greater. Of note, prior to January 2019 a cycle threshold value of 36 or greater was considered positive in Missouri, and prior to May 2015 a TREC level of 300 copies/μL or less was considered positive in Illinois. Cycle thresholds greater than 39 or TREC levels less than 25 copies/μL were considered high risk and resulted in immediate referral for further workup instead of repeating the newborn screen.

Data for our study population was compiled from IRB-approved retrospective electronic medical record review of infants referred between July 2014 and January 2018 and a prospective primary immunodeficiency database of patients from January 2018 until March 2020. We excluded premature patients if their positive screen normalized when repeated after 36 weeks corrected gestational age. Demographic information, relevant clinical history, and laboratory work from their initial clinical evaluation and follow up visits were reviewed. Laboratory studies from the initial evaluation included CBC with differential, quantitation of lymphocyte subpopulations, TREC copy number analysis normalized to CD3^+^ T cell count, absolute CD4RTE (recent thymic emigrant, defined as CD4^+^ CD45RA^+^ CD31^+^ T cells), and naïve Th cell percentage (defined as percentage of CD4^+^ CD45RA^+^ cells out of the total CD4^+^ cells). Results of chromosome microarray, targeted gene panels (GeneDx^®^ SCID panel or Invitae^®^ Primary Immunodeficiency panel), and whole exome sequencing were also reviewed if obtained for clinical care. Diagnostic outcomes were classified into 3 groups including 1) severe T cell deficiency due to SCID and complete DiGeorge phenotype, 2) non-SCID TCL either due to known genetic or secondary causes or idiopathic, and 3) normal T cell count. For the first category, SCID was further characterized as typical or leaky SCID. Typical SCID was defined as having CD3^+^ T cells < 300 cells/μL, less than 10% of the lower range of normal proliferation to phytohemaglutinin, and/or detectable maternal T cell engraftment. Leaky SCID was defined as having a CD3^+^ T cell count of >300 cells/μL, but with a restricted TCR repertoire and/or lack of naive T cells (3). For the second category, non-SCID TCL was defined as having CD3^+^ T cells < 2,500 cells/μL, and further classified as mild (CD3^+^ T cells >1,500-2,500 cells/μL) or moderate (CD3^+^ T cells 300-1,500 cells/μL). Idiopathic TCL was defined as non-SCID TCL without a known underlying genetic or secondary cause, in some cases based on negative chromosome microarray, whole exome sequencing, and/or targeted genetic testing (3, 8).

Statistical analysis was performed to identify correlations between CD3^+^ T cell count, TREC, CD4RTE, naïve Th cell percentage, and absolute lymphocyte count (ALC), and to determine the differences in these variables between the normal T cell count and non-SCID TCL groups. Data is presented as mean ± standard deviation and range for quantitative variables. Pearson’s correlation coefficient (ρ) was calculated to assess correlations between two continuous lab values. Paired T-test was used to assess the differences in lab values between normal and non-SCID TCL groups. Data was analyzed by SAS^®^ (SAS Institute Inc., Cary, NC, USA) 9.4 version. A P-value < 0.05 was considered statistically significant.

## Results

**Figure 1:**
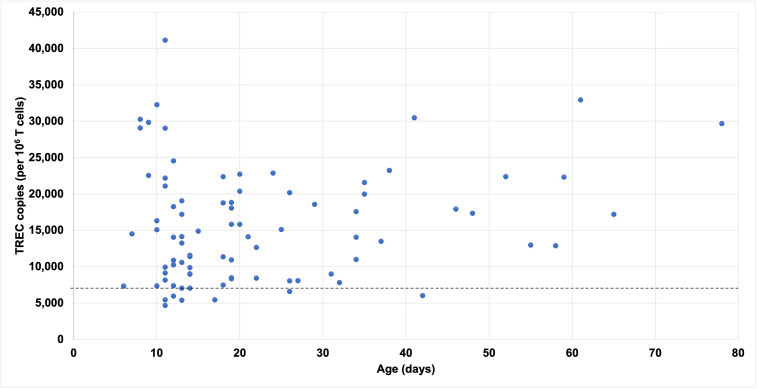
TREC copies by age at the time of testing in term infants with abnormal SCID screens and normal CD3^+^ T cell count. Dashed line represents normal cutoff TREC value for term infants.

A total of 154 infants were included in the study as shown in **Table 1**. Of these, 72% were male, 62% were Caucasian, and 33% were African-American. 51% were from Missouri and the rest from Illinois. 3% were premature but had persistently abnormal newborn SCID screening at 36 weeks corrected gestational age or later. The mean age at initial evaluation was 22 days (1-86 days). 60% of infants had a normal CD3^+^ T cell count (N=91) or normal repeat newborn screen (N=2), and their repeat TREC copy number analysis normalized to CD3^+^ T cell count was normal or near-normal in all 84 infants tested (**Figure 1**). Prior to January 2019, when Missouri’s cycle threshold cutoff for a positive SCID screen was increased, this false positive rate was slightly higher at 69%. 33% of infants had non-SCID TCL (mild, N=26 and moderate, N=25), and 6% had typical SCID (N=6), leaky SCID (N=3) or complete DiGeorge phenotype (N=1).

**Table 1:** Demographic and diagnostic characteristics of infants with positive newborn SCID screens.

Demographic characteristic (N=154)	N (%)
Male	111 (72)
Race	
White	95 (62)
Black	51 (33)
Asian	1 (0.6)
Other/Unknown	7 (5)
States where newborn screening obtained	
Illinois	76 (49)
Missouri	78 (51)
Pre/peri-natal complications	54 (35)
Mean age at initial evaluation	22 days
Diagnostic categories	
*Normal findings*	93 (60)
No T cell lymphopenia	74 (48)
Normal CD3 but other cell lines abnormal	17 (11)
Repeat newborn screen normal	2 (1)
*Non-SCID TCL*	51 (33)
Non-SCID TCL with genetic or secondary cause	26 (17)
Idiopathic non-SCID TCL	25 (16)
*SCID and other severe TCL*	10 (6)
Typical SCID	6 (4)
Leaky SCID	3 (2)
Complete DiGeorge	1 (0.6)

### Causes of Non-SCID T-Cell Lymphopenia and Their Outcomes

Twenty-six infants had non-SCID TCL with a known genetic or secondary cause, of which 20 had moderate and 6 had mild TCL. All had follow-up visits at a median age of 14 months (10 days – 59 months) except for 1 patient with trisomy 21 for whom immunology follow up was recommended as needed. Genetic etiologies included 22q11 deletion (N=16), trisomy 21 (N=2), and genetic variants presumed to be pathogenic in *TBX1* (N=2), *FOXN1* (N=1), *MYSM1* (N=1), *CD3E* (N=1), *ATM* (N=1), and *POLD1* (N=1). Three infants with 22q11 deletion died, including 1 preterm infant who died from necrotizing enterocolitis, and 2 term infants who had complicated post-operative courses and cardiac arrest following surgery for their congenital heart disease. Six infants, all with 22q11 deletion, had recurrent infections or hospitalization for infections, and 1 patient has required treatment with immunoglobulin replacement therapy and antibacterial prophylaxis.

All patients in our cohort with monogenic variants felt to be likely pathogenic are summarized in **Table 2**, including 5 patients with novel variants associated with non-SCID TCL. P1 had progressively worsening pancytopenia requiring hematopoietic stem cell transplant and ultimately passed away from hepatic veno-occlusive disease in the post-transplant period. P2 and P3 had improving or relatively stable T cell counts over time and no significant infections. P5 had 2 pathogenic bi-allelic variants for which his parents were found to be heterozygous carriers, thereby confirming his diagnosis of ataxia-telangiectasia associated with T and B cell lymphopenia. While his newborn screen showed a mildly low level of 217.6 TREC copies/μL, his identical twin brother had a normal screen with 368.8 TREC copies/μL. The twin brother also had the same pathogenic variants, T and B cell lymphopenia, and elevated AFP level confirming the diagnosis of ataxia-telangiectasia. P6 had a heterozygous variant of uncertain significance that was absent in both her parents. This patient had abnormal DNA repair based on radiosensitivity testing at 6 weeks age. In addition to lymphopenia, she had anemia requiring transfusions in the first month of life. She was started on immunoglobulin replacement and antimicrobial prophylaxis and has not had any infections. At 17 months, her radiosensitivity testing normalized, and her CD4^+^ T cell count remains low but has improved from 398 cells/μL at 2 weeks of age to 1083 cells/μL at 17 months. P7 had improving T cell count and her TCL resolved at 11 months of age. She was doing well without infections.

**Table 2:** Monogenic variants detected in patients with non-SCID TCL and SCID or other severe TCL.

Patient	Coordinates (Build hg38)	Gene	Coding change	Protein change	Zygosity and inheritance if known	CADD	Allele Frequency in population (Gnomad)	Immune phenotype
**Non-SCID TCL**								
P1	1:58667227	MYSM1*^†^	c.1843-1G>A (NM_001085487.2)	IVS15-1G>A	Homozygous; paternal uniparental disomy of chromosome 1	33	N/A	Pancytopenia
P2	17:28530824	FOXN1*^†^	c.907delG (NM_003593.2)	p.Glu303Serfs*247	Heterozygous; maternal	33	N/A	Isolated TCL
P3	22:19763331	TBX1*	c.498_501dupCGAT (NM_080647.1)	p.Lys168ArgfsX2	Heterozygous; paternal	33	N/A	Isolated TCL
P4	22:19765069	*TBX1*	c.796G>T (NM_080647.1)	p.Glu266Ter	Heterozygous	41	N/A	Isolated TCL
P5	11:108257471	*ATM*	c.2251-10T>G	Intronic	Heterozygous; maternal	31	0.000006569	T and B cell lymphopenia
	11:108301698		c.5228C>T (NM_000051.3)	p.Thr1743Ile	Heterozygous; paternal	25.9	0.000006576	
P6	19:50409228	POLD1*^†^	c.1999C>T (NM_002691.3)	p.Arg667Trp	Heterozygous; *de novo*	27	N/A	T and B cell lymphopenia
P7	11:118315498	CD3E*^†^	c.580G>A (NM_000733.3)	p.Gly194Ser	Heterozygous	29.8	0.00009880	Isolated TCL
**SCID**								
P8	19:17841764	JAK3*	c.862-2A>G	IVS6-2A>G	Heterozygous	34	N/A	T-B+NK+
	19:17843162		c.431A>T ^†^ (NM_000215.3)	p.Asp144Val	Heterozygous	25.9	0.000006571	
P9	19:17843843	JAK3*	Exon 10 deletion	N/A	Heterozygous		N/A	T-B+NK-
			c.242G>T (NM_000215.3)	p.Trp81Leu	Heterozygous	26.3	N/A	
P10 and P11 (Siblings)	20:44623039	*ADA*	c.646G>A (NM_000022.2)	p.Gly216Arg	Homozygous	27.4	0.00005257	T-B-NK-
P12	X:71110537	*IL2RG*	c.421C>T (NM_000206.2)	p.Gln141Ter	Hemizygous	33	N/A	T-B+NK+
P13	X:71110567	*IL2RG*	c.391C>T (NM_000206.2)	p.Gln131Ter	Hemizygous	35	N/A	T-B+NK-
**Other severe TCL**								
P14	11:36573831	*RAG1*	c.527G>T (NM_000448.2)	p.Cys176Phe	Homozygous; maternal and paternal	28.1	0.00001972	T-B+NK+ leaky SCID
P15	8:60821779	CHD7*^†^	c.2698-11A>G (NM_017780.3)	Intronic	Heterozygous	16.66	N/A	CHARGE syndrome and complete DiGeorge phenotype with absent T cells, low B cells, low NK cells
	9:35657772	*RMRP* ^†^	r.247G>C (NR_003051.3)	N/A	Heterozygous	22.3	N/A	

*Novel variant at time of diagnosis. ^†^Variant of uncertain significance.

One patient had a secondary cause of non-SCID TCL due to congenital thoraco-cervical fibrosarcoma who underwent resection of the mass in the immediate postnatal period. His CD3^+^ T cell counts significantly improved over time from 393 cells/μL initially to 1849 cells/μL, suggesting that mass effect from the fibrosarcoma had impaired his thymic development, resulting in a DiGeorge-like phenotype.

Twenty-five infants in our study had idiopathic non-SCID TCL, of which 5 had moderate and 20 had mild TCL, and all had normal CD3^+^ T cell proliferative responses to phytohemagglutinin and pokeweed. Six infants were lost to follow up, 1 transferred care to a different institution, and 2 did not require follow up. Among the 16 patients that did follow up at a median age of 5 months (2.5-26 months), 7 had a normal chromosome microarray or FISH testing for DiGeorge syndrome and 3 had negative SCID genetic testing. Only 2 infants with idiopathic TCL had recurrent infections – 1 with recurrent ear infections that improved after tympanostomy tube placement, and 1 with recurrent upper respiratory infections prompting a chromosome microarray to rule out partial DiGeorge syndrome. This showed a 1q21.1 microduplication that was not felt to be related to his lymphopenia. Additionally, 1 infant had a hospitalization for periorbital cellulitis requiring 1 day of IV antibiotics, and another had a PICU admission for RSV infection without bacterial superinfection. None of the infants with idiopathic TCL required antimicrobial prophylaxis or immunoglobulin replacement.

**Figure 2:**
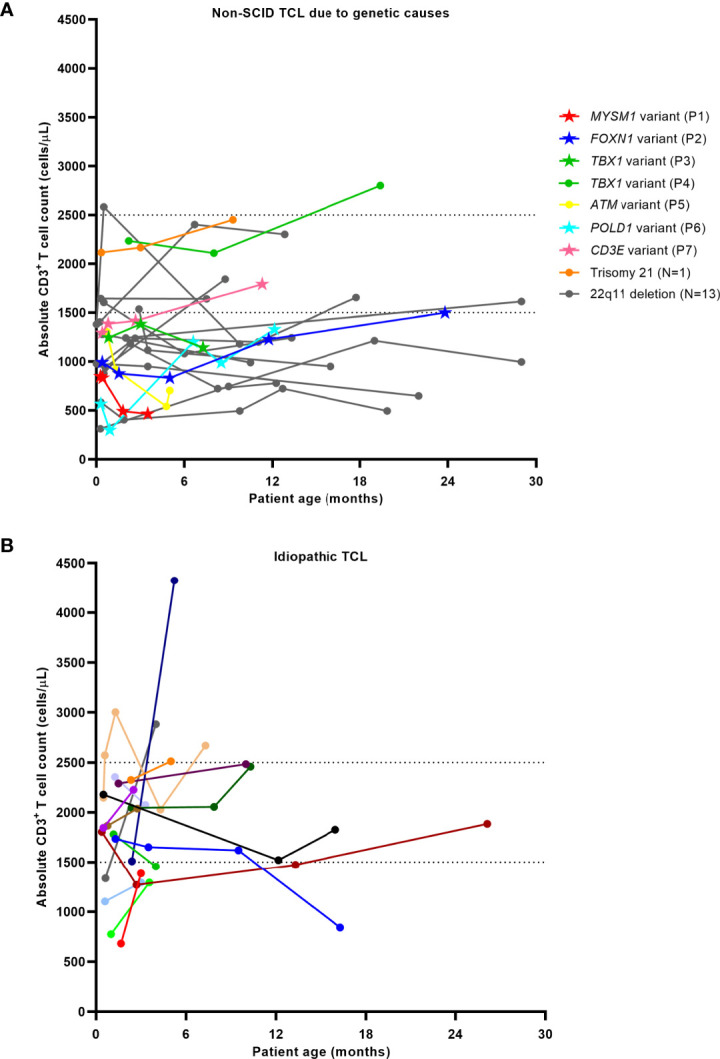
T lymphocyte trends in patients with non-SCID TCL due to genetic causes **(A)** and idiopathic TCL **(B)**. Dashed lines indicate cutoffs for mild (1500-2500 cells/μL) and moderate (300-1500 cells/μL) TCL. Data sets represented by stars in **Figure 1A** indicate the patients with novel pathogenic variants at the time of diagnosis.

Individual T lymphocyte trends of non-SCID TCL infants from the time of initial evaluation until as late as 30 months age are shown in **Figure 2**. Among infants with non-SCID TCL with a known genetic or secondary cause, T cell counts rose in 50% of infants from an average of 1133 ± 626 cells/μL to 1778 ± 544 cells/μL and dropped in the other 50% of infants from an average of 1206 ± 478 cells/μL to 930 ± 357 cells/μL. Among infants with idiopathic TCL, T cell counts rose in 75% of infants from an average of 1644 ± 564 cells/μL to 2288 ± 841 cells/μL and dropped in the other 25% of infants from an average of 2013 ± 304 cells/μL to 1550 ± 535 cells/μL. Four of the infants that improved had normalization of T cell count to 2500 cells/μL or greater by a median age of 5.1 months (4-7.3 months).

### SCID Screening Outcomes in DiGeorge Syndrome

**Figure 3:**
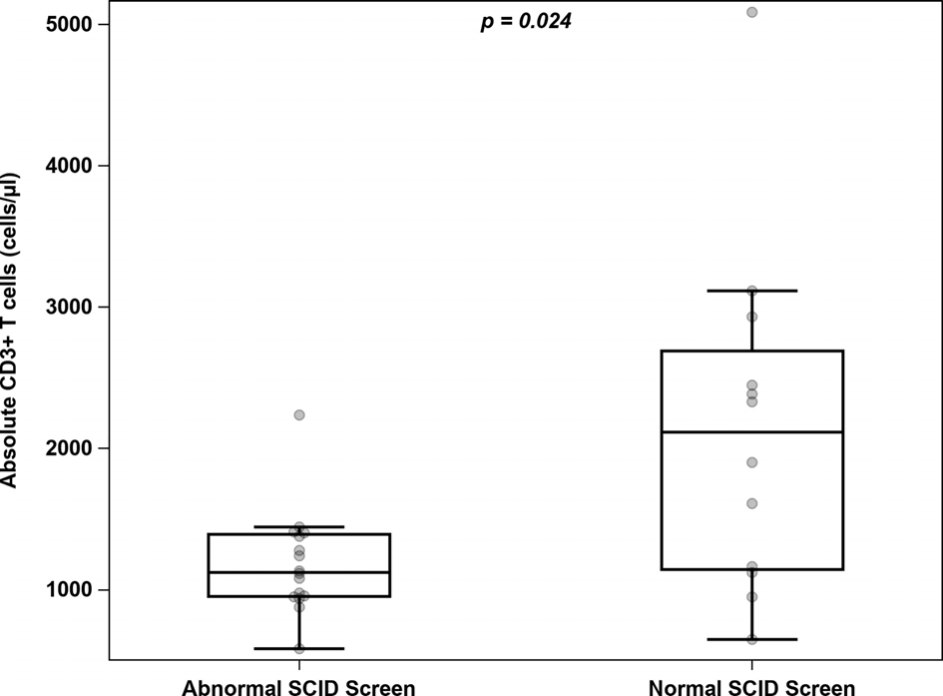
Distribution of absolute T cell counts among infants with 22q11 deletion and either abnormal or normal newborn SCID screening.

During the 6-year study period, our institution cared for a total of 28 infants with DiGeorge syndrome (DGS) including 26 infants with 22q11.2 deletion and 2 infants with heterozygous pathogenic variants in *TBX1* who had undergone newborn SCID screening (**Table 3** and **Figure 3**). Sixteen infants (57%) had an abnormal SCID screen, and those patients had a lower median CD3^+^ T cell count compared to infants with normal SCID screens: 1124 (954-1392) cells/μL *vs.* 2116 (1143-2690) cells/μL respectively, p=0.024. TCL was present in a higher percentage of the infants with abnormal screens than those with normal screens: 94% *vs.* 33% respectively, p=0.001. However, the age at blood draw for the first T cell count was different between the groups, with a median age of 12 days (range 1-86 days) in DGS infants with abnormal SCID screen and a median age of 90 days (range 7-367 days) in DGS infants with normal SCID screen. Four of them were brought to medical attention solely based on the abnormal newborn screen, including 1 patient who was later found to have significant congenital heart disease requiring life-saving intervention.

**Table 3:** Results of newborn SCID screening in infants with 22q11 deletion syndrome diagnosed during the 6-year study period.

Parameters	Total infants with 22q11 deletion (N=28)		P-value
	Abnormal SCID screen (N=16)	Normal SCID screen (N=12)	
Median CD3^+^ T cells/μL (IQR)	1124 (954-1392)	2116 (1143-2690)	0.024
Number of infants with CD3^+^ T cells < 1500 (%)	15 (94%)	4 (33%)	0.001

Of the 12 DGS patients with normal SCID screen, clinical presentations that led to DGS diagnosis were congenital heart diseases (n=6), failure to thrive and feeding difficulty (n=2), prenatal diagnosis due to abnormal fetal ultrasound (n=2), congenital diaphragmatic hernia (n=1), and developmental delay (n =1). After exclusion of two patients who were prenatally diagnosed, the age at DGS diagnosis in infants with normal SCID screen ranged from 3 days to 10 months, with a median age of 39 days. Of 16 DGS patients with abnormal SCID screen, after exclusion of 5 infants who were prenatally diagnosed, the age at DGS diagnosis ranged from 2 to 95 days, with a median age of 30 days.

### Diagnostic Outcomes for SCID and Comparison of Outcomes Before/After Screening

Six infants were diagnosed with typical SCID by newborn screening due to pathogenic variants in *JAK3* (N=2), *ADA* (N=2), and *IL2RG* (N=2) as listed in **Table 2**. Two patients with ADA deficiency were siblings, and genetic testing was not done on the younger sibling; however, he had an undetectable ADA1 enzyme level confirming the diagnosis ADA deficiency. The ALC ranged from 100 to 880 cells/μL (median of 450 cells/μL) in these 6 infants. All underwent HSCT at a median age of 5 weeks (3 weeks – 4 months). All patients had full engraftment of donor T cells at 6 months and 1 year post-transplant, with the exception of patient P10 who required 2 repeat transplants as a result of graft failure by 6 months following the initial transplant. None of the patients had infections prior to transplant, with the exception of P11, a patient with *ADA*-SCID (sibling of P10) who had two observational hospitalizations for rhino/enterovirus and parainfluenza upper respiratory infection, neither of which required any respiratory support. In contrast, the 4 infants diagnosed with typical SCID at our institution prior to newborn SCID screening (2008 – 2014) had a median age of 13 months (4-22 months) at transplant (**Table 4**). Three had successful engraftment of donor T cells at 6 months and 1 year post-transplant, but two patients required repeat transplants at 2 and 7 years of age due to poor immune reconstitution and T cell exhaustion, respectively. The fourth patient had failed engraftment at 6 months requiring a repeat transplant. All patients had infections at initial presentation.

**Table 4:** Transplant outcomes for SCID and leaky SCID at SLCH 2015-present.

Parameters	Before newborn screening		After newborn screening	
	Typical SCID (N=4)	Leaky SCID (N=1)	Typical SCID (N=6)	Leaky SCID (N=4*)
Median age at diagnosis	10 months (3-19 months)	1 month	9 days (5-18 days)	13 days (2-28 days)
Genetic etiology	IL2RG (2), PNP (1), RMRP (1)	RAG1	JAK3 (2), IL2RG (2), ADA (2)	RAG1 (2), negative genetic workup (2)
Median age at transplant	13 months (4-22 months)	3 months	5 weeks (3 weeks – 4 months)	6 months (5-6 months)
Median age at most recent follow-up	5 years (4-7 years)	11 months	3 years (9 months – 5 years)	17 months (9 months – 3 years)
Outcomes	Engrafted at 6 months and 1 year (1), engrafted after 1 repeat transplant (1), engrafted at 6 months and 1 year but required repeat transplant >1 year later (2)	Engrafted at 6 months but died later of unclear etiology	Engrafted at 6 months (1), engrafted at 6 months and 1 year (4), engrafted after 2 repeat transplants (1)	Engrafted at 6 months (1), engrafted at 6 months and 1 year (1), engrafted after 1 repeat transplant (1), deferred transplant and died from sepsis (1)

Engraftments at 6 months and 1 year post-transplant, if available, were assessed. Repeat transplants were performed following failed engraftment at 6 months after the initial transplant or at later time points if indicated below.

*One infant with RAG1 deficiency who was initially managed at an outside institution prior to his transplant. A patient with IL2RG deficiency who was missed from newborn screening was not included as an outlier.

Three infants in our study were diagnosed with leaky SCID. Their ALC ranged from 600 to 1300 cells/μL. P14 had a homozygous *RAG1* pathogenic variant as well as *POMT1-*associated muscle-eye-brain disease. In this case, the parents elected to postpone transplant due to underlying neurological condition. She died at 17 months from sepsis associated with ventriculoperitoneal shunt infection. Another patient had unrevealing genetic workup including whole exome sequencing and a targeted SCID genetic panel, which showed heterozygous variants of unknown significance in *DOCK2* and Artemis that are typically associated with autosomal recessive disease. However, he had abnormal radiosensitivity testing and persistent lymphopenia, and underwent HSCT at 6 months of age with full engraftment at 6 months post-transplant. The third infant had negative genetic workup including chromosome microarray, clinical whole exome sequencing, and targeted SCID genetic testing. Research-based T cell development testing suggested intrinsic hematopoietic abnormalities (13), and he underwent HSCT at 5 months of age, with full engraftment at 1 year post-transplant. One infant with leaky SCID who is not included in the total study population of 154 infants was found to have an *IL2RG* mutation despite a normal newborn SCID screen (14). It is challenging to compare outcomes in patients with leaky SCID before and after implementation of SCID screening as we had only one patient with leaky SCID prior to implementation of SCID newborn screening.

Patient P15 with CHARGE syndrome and complete DiGeorge phenotype underwent thymic transplant, however none of the other patients with primary thymic defects such as P2, P3, or P4 had severe T cell lymphopenia or significant T cell dysfunction to require a thymic transplant.

### Correlations Between Parameters Measuring Thymic Output

**Figure 4:**
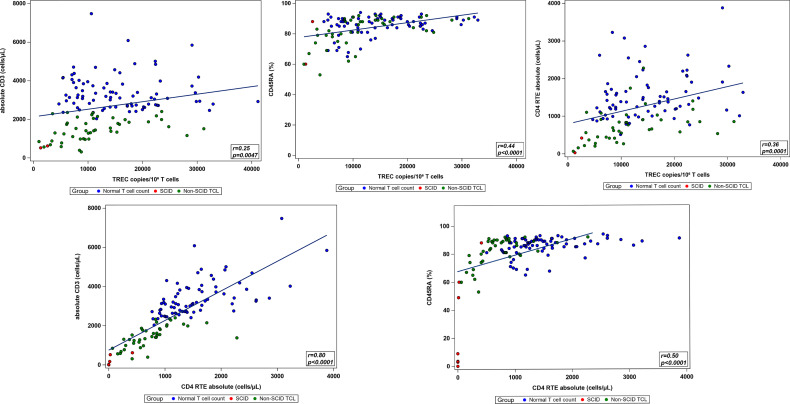
Pairwise correlations between immunologic parameters of interest.

Pairwise correlations between the total CD3 count, TREC copy number normalized to CD3 count, absolute CD4RTE, naïve Th cell percentage, and ALC were calculated using the total study population, excluding patients with undetectable or 0 TREC copies (**Table 5** and **Figure 4**). All correlations were significantly positive, except for that between ALC and TREC copies, and the strongest correlation was between CD4 RTE and CD3 count (ρ=0.80, p<0.0001). Significant moderate positive correlations were found between TREC copies and naïve Th cell percentage and between CD4 RTE and naïve Th cell percentage, while significant weak positive correlations were found between TREC copies and CD3 count and between TREC copies and CD4RTE. ALC correlated strongly with CD3 count and moderately with CD4RTE and naïve Th cell percentage. When comparing the normal T cell count, non-SCID TCL with known genetic or secondary cause, and idiopathic TCL groups, significant differences in all lab parameters were found between the non-SCID TCL with known genetic cause and normal groups, as shown in **Table 6**. However, only CD4RTE and ALC were significantly different between the idiopathic TCL and normal groups.

**Table 5:** Pairwise correlations between immunologic parameters of interest.

Correlation	Pearson’s correlation coefficient	P-value
TREC copies and absolute CD3	0.25	0.0047
TREC copies and naïve Th cell percentage	0.44	<0.0001
TREC copies and CD4RTE	0.36	0.0001
CD4 RTE and absolute CD3	0.80	<0.0001
CD4 RTE and naïve Th cell percentage	0.50	<0.0001
ALC and absolute CD3	0.75	<0.0001
ALC and TREC copies	0.17	0.0612
ALC and CD4RTE	0.59	<0.0001
ALC and naïve Th cell percentage	0.45	<0.0001

Patients with undetectable or 0 TREC copies were excluded from the correlations.

**Table 6:** Comparison of immunologic parameters of interest between infants with normal T cell count, non-SCID TCL due to genetic or secondary causes, and idiopathic TCL.

Lab value	Normal (N=88)	Non-SCID TCL due to genetic or secondary cause (N=26)	Idiopathic TCL (N=25)
TREC copies per 10^6^ T cells	15548.14 ± 7800.03	8406.48 ± 3733.40	14296.48 ± 8058.81
		P = 0.0001	P = 0.49
CD4 RTE (cells/μL)	1547.2 ± 631.35	598.84 ± 497.59	850.88 ± 363.51
		P < 0.0001	P < 0.0001
Naïve Th cell percentage	85.73 ± 6.53	79 ± 12.13	84.21 ± 6.23
		P = 0.0014	P = 0.32
ALC (10^3^ cells/μL)	4.8 ± 1.5	2.7 ± 1.5	3.2 ± 0.9
		P < 0.0001	P < 0.0001

P-values reflect comparison with the normal T cell count group.

### Perinatal Complications and Birth Weight

Roughly a third of all patients (N=54) had a prenatal or perinatal complication. The most common were neonatal respiratory distress syndrome or oxygen requirement (N=16), either chronic or gestational maternal diabetes (N=13), maternal hypertension or pre-eclampsia (N=10), and intrauterine recreational drug exposure (N=8). Four infants were born to mothers who had received betamethasone for threatened preterm labor, otherwise none had been exposed to any other immunosuppressant medications *in utero*. Three of these patients had normal CD3^+^ T cell counts, including 1 with B cell and CD4 lymphopenia, and the fourth had non-SCID TCL due to partial DiGeorge syndrome. There was no significant difference in initial CD3 count (2668 ± 1186 *vs.* 2452 ± 1387 cells/μL, p=0.35) or total TREC copies (13385 ± 7431 *vs.* 13809 ± 8360 copies/10^6^ T cells, p=0.77) between infants with complications and those without. The incidence of any form of TCL was 37% among infants with complications and 41% among those without.

Birth weights were available from chart review for 139 of the 154 total patients. Of these, 20 (14%) were characterized as low birth weight at less than 2500 grams. Comparison between low and normal weight infants showed no significant differences in CD3 counts (2706 ± 1241 *vs.* 2477 ± 1341 cells/μL, p=0.43) or TREC copies (13704 ± 9520 *vs.* 14331 ± 8031 copies/10^6^ T cells, p=0.81). Additionally, both groups had the exact same prevalence of TCL of 40%.

## Discussion

Our study reviewed the immunologic phenotypes of 154 infants with positive SCID newborn screens referred from both Illinois, where SCID screening started in 2014, and Missouri, where it started in 2017. These infants represent a third of total Missouri referrals and an unknown portion of Illinois referrals, with choice of referral site determined by patient/family and primary care provider preference. Over a total 6-year period, SCID screening detected CD3^+^ TCL of varying degrees in 39% of the study population, including 33% with non-SCID TCL and 6% with SCID or other severe forms of TCL. Although our institution receives only a third of the total statewide referrals for abnormal newborn screens, these rates are consistent with the unpublished statewide data from Missouri in 2019 which showed that out of 13 total abnormal newborn SCID screens that year, 5 (38%) were due to non-SCID TCL and 1 (7.7%) due to SCID. In comparison, the positive predictive value of newborn screening for TCL was 48% in Wisconsin (15), 38% in California (5), and 20.3% in New York (16). The variation in these rates can be attributed to the differences in screening algorithms and testing thresholds for TCL between different states (2). As expected, the positive predictive value of SCID newborn screens at our institution was higher prior to 2019 when Missouri’s cycle threshold cutoff was lower.

Of note, three quarters of infants referred to our center in this study were male, suggesting an increased prevalence of TCL in males compared to females, the reason for which is unclear though male sex has previously been associated with low TREC (17). A similar trend has been seen in multiple other states, where the proportion of males among infants with TCL ranged from 59 to 76% (2). Furthermore, our study excluded premature infants whose newborn screens normalized after 36 weeks, so the increased incidence of prematurity among males compared to females would not be expected to influence our results. The proportion of African-American infants in our study population (33%) was also higher than in the general population. This was observed in New York state as well where Vogel et al. found that 41.6% of infants with positive newborn SCID screens were African-American (16). However, a larger study from California showed no association between ethnicity and newborn SCID screen outcome (3). Therefore, further studies will be needed to determine whether ethnic differences in neonatal thymic function and lymphopoiesis exist that could be a risk factor for having a positive newborn screen.

As with any form of population wide screening, the newborn SCID screen has revealed significant phenotypic diversity, especially in its detection of non-SCID TCL. In our study group, the screening led to identification of novel genetic variants in *FOXN1*, *TBX1*, *MYSM1*, *POLD1*, and *CD3E* associated with non-SCID TCL. To our knowledge these variants had not been previously reported in the literature at the time of diagnosis. *FOXN1* plays a regulatory role in thymic epithelial cell development, such that *FOXN1* haploinsufficiency has been shown to result in neonatal TCL that gradually improves with age (18). This has so far been the case for our patient with the *FOXN1* variant (P2). Loss-of-function variants in *TBX1* have previously been reported in patients with DiGeorge syndromic features and TCL, but without 22q11 deletion (19). However, our patient with a *TBX1* variant (P3) did not have any physical features of velocardiofacial syndrome, only diminished lymphopoiesis. P4 on the other hand also had a TBX1 variant but had congenital heart defects and TCL that later normalized. The patient with a novel *MYSM1* variant (P1) suffered worsening pancytopenia and unfortunately died from post-transplant complications. This gene encodes a histone deubiquitinase that controls expression of transcription factors involved in hematopoiesis and lymphocyte differentiation. Homozygous missense and nonsense variants leading to *MYSM1* deficiency have previously been reported in patients presenting with bone marrow failure and B cell lymphopenia (20, 21).

Some of the infants in our study who had syndromes associated with non-SCID TCL presented to medical attention solely based on their abnormal newborn SCID screens, highlighting the ability of SCID screening to facilitate earlier diagnosis and management of these conditions. For example, 22q11 deletion was the most common genetic cause for non-SCID TCL, and a quarter of the infants with this diagnosis in our cohort had no other characteristic findings of the disease besides TCL detected by newborn screen. One of these patients was subsequently diagnosed with a significant congenital heart defect, which might not have otherwise been detected in a timely manner allowing for treatment. However, the 57% positivity rate of newborn SCID screening for infants with DiGeorge syndrome at our institution indicates that a normal newborn screen does not exclude DiGeorge syndrome. Since our institution’s DiGeorge syndrome population primarily includes patients with associated complications such as congenital heart disease who require hospital based care, the DiGeorge syndrome population as a whole including undiagnosed individuals with milder features would likely have a lower SCID screen positivity rate than observed in our study. Additionally, it is to be determined in a larger cohort if SCID newborn screening impacts long-term outcomes of incomplete DiGeorge syndrome patients as the data from our cohort showed no difference in median age at diagnosis between DiGeorge syndrome infants with abnormal SCID screens and normal SCID screens. Our findings also suggest infants with DiGeorge syndrome and abnormal SCID screens had lower T cell counts compared to infants with DiGeorge syndrome and normal SCID screens. However, this is likely confounded by a later age at immune evaluation in infants with normal SCID screens.

Another example of earlier disease diagnosis due to detection of non-SCID TCL is the infant diagnosed with ataxia-telangiectasia at 6 months age (P5) as a result of workup for his B and T cell lymphopenia that were uncovered by follow-up testing for an abnormal newborn screen. The diagnosis was made prior to the onset of any other disease manifestations, and allowed for early diagnosis of his identical twin brother as well, who interestingly had a normal newborn screen TREC value. The diagnosis of ataxia-telangiectasia by newborn SCID screening was also demonstrated in 4 patients in Ontario, Canada, and shown to be associated with more severe immunologic and neurologic involvement as compared to individuals diagnosed later in life. However, earlier diagnosis also prompted earlier referral to other specialty services as well as family genetic counseling and screening for malignancies in the carriers of the *ATM* variants (22). Given that there is no definitive cure at this time for ataxia-telangiectasia, the overall benefit of early diagnosis remains controversial. However, these cases illustrate the ability of newborn SCID screening to detect not only SCID, but other conditions associated with TCL earlier than they might otherwise be diagnosed. This has the potential to allow for appropriate interventions to be instituted prior to the onset of other disease complications.

Longitudinal follow up of idiopathic TCL in our study revealed favorable outcomes overall. T cell counts rose over time in 75% of infants, and even normalized in 25% at the median age of 5 months (range 2.5 to 26 months). These patients had fewer infectious complications compared to those with a known genetic cause of non-SCID TCL. Additionally, none of the infants with idiopathic TCL required antibiotic prophylaxis or immunoglobulin replacement. These findings are reassuring as idiopathic TCL remains a diagnosis of exclusion, and one for which the optimal monitoring, management strategies and long-term prognosis are still being defined. In the future, more extensive genetic testing of these infants may reveal new etiologies for lymphopenia, allowing for less testing and monitoring in the cases where there are no long-term consequences of TCL. Our study was limited by the fact that half of infants with idiopathic TCL did not undergo genetic testing, as it was not considered clinically indicated given the milder degree of lymphopenia in these infants and the positive trend in their T cell counts over time. Additionally, only 13 of 51 infants with non-SCID TCL were inpatients at the time of initial evaluation. The fact that the majority of patients recruited in our study were outpatients could potentially explain the lower incidence of secondary causes of TCL in our cohort compared to prior studies, since patients with lymphopenia due to conditions like gastroschisis, thymectomy, or other secondary causes would be more likely diagnosed in the inpatient setting.

Infants diagnosed with typical SCID by newborn screening benefited from earlier diagnosis and improved transplant outcomes compared to those diagnosed before the implementation of routine screening (median age at diagnosis of 9 days *versus* 10 months, respectively). Three out of the 4 infants diagnosed prior to newborn SCID screening required repeat transplants, while only 1 of the 6 diagnosed after newborn screening required a repeat transplant, suggesting greater success of transplant when performed at an earlier age and prior to the onset of opportunistic infections that were encountered by the infants diagnosed prior to newborn SCID screening. This finding is consistent with previously published outcomes in transplant for SCID. In our cohort, differences may also be influenced by differences in duration of follow up between the two groups (median age at most recent follow up of 5 years *versus* 3 years in the groups diagnosed before and after routine screening, respectively), however the rate of graft failure at 6 months post-transplant was also greater prior to newborn SCID screening (1 out of 4) than afterward (1 out of 6). Although nearly all infants with leaky SCID were diagnosed within the first month of life by newborn screening, they were transplanted at later ages than infants with typical SCID due to longer times to establish definitive genetic diagnosis or evidence of continued T cell decline and evident hematopoietic defect. One patient with *RAG1* mutation was delayed due to logistics related to transfer from outside institution. One patient had no identified genetic defect and proceeded to transplant only after continued decline in T cells and evidence of hematopoietic defect based on research studies at National Institutes of Health.

The cutoff levels for a high-risk abnormal newborn screen – cycle threshold greater than 39 in Missouri or TREC level less than 25 copies/μL in Illinois – were highly specific; only 1 out of the 21 infants with high-risk screens in our cohort had a normal immune workup. However, one of the patients with leaky SCID due to *IL2RG* mutation was missed by newborn screening (14), a reminder that SCID screening is not 100% sensitive. Infants with false-positive newborn screens had normal TREC copy number analysis normalized to CD3^+^ T cell count, which may be explained by differences in TREC cutoff values for TREC quantitation and limitations related to using dried blood spot samples, although both methods involve real-time PCR. Whereas the newborn screen reflects the TREC copies per microliter of blood in the dried blood spot specimen, the repeated TREC analysis reflects TREC copies per million cells in a purified CD3^+^ T cell population and therefore is more specific.

The laboratory workup of infants referred for positive newborn SCID screens at our institution allowed for the observation that certain immunologic parameters had stronger positive correlations than others. When comparing TREC copies normalized to CD3 count and CD4RTE, CD4RTE had a stronger positive correlation with the CD3 count and naïve Th cell percentage; however, all were statistically significant correlations. Therefore, this finding does not diminish the utility of TREC based screening for assessment of T cell count in newborns, but provides support for considering CD4RTE as part of the evaluation of an infant with a positive newborn SCID screen. In addition, the comparison of these lab parameters between patient groups showed greater differences in TREC copies, CD4RTE, and naïve Th cell percentage between the non-SCID TCL with known genetic cause and normal groups than between the idiopathic TCL and normal groups. Despite the mildly decreased net thymic output in idiopathic TCL, these infants demonstrated relatively normal T cell function as they generally did not develop recurrent or severe infections.

Despite a third of infants in the study having a prenatal or perinatal complication, there were no significant difference in T cell counts or TREC copies between these infants and those without complications. Similarly, no significant differences were seen in these parameters between low birth weight infants and normal weight infants. However, this is limited by the fact that only 14% of the infants in the study had low birth weight. Since the association between birth weight and TRECs is more pronounced with lower gestational age, the exclusion of premature infants in our study may also obscure this relationship (23).

We acknowledge several limitations of our study. Our findings represent only a subset of infants with abnormal SCID screen from Illinois and Missouri during the study period and, as mentioned above, we were unable to assess the total number of infants screened in both states during the study period. Most patients were recruited outpatient which led to an underestimated number of patients with TCL from secondary causes. The small-size cohort also limited the ability to assess the significance of other factors including prenatal/perinatal complications and birth weight that may affect TREC levels.

In conclusion, our study adds to the current literature on newborn SCID screening by demonstrating novel genetic variants associated with non-SCID TCL and identifying correlations between various measures of thymic output that may guide the future laboratory workup and interpretation of infants with positive SCID screening. SCID newborn screen detected approximately half of patients with DiGeorge syndrome during neonatal period. It also strengthens and adds to the observation that idiopathic TCL, as opposed to non-SCID TCL with known genetic or secondary causes, generally has a benign clinical course by showing lower incidence of infections, greater chance of rising T cell count over time, and greater degree of similarity with healthy infants in terms of TREC and naïve T cell production. As newborn SCID screening undoubtedly continues to reveal individuals with non-SCID TCL, our understanding of T cell development and the numerous factors that influence it will continue to grow.

## Data Availability Statement

The original contributions presented in the study are included in the article/supplementary files. Further inquiries can be directed to the corresponding author.

## Ethics Statement

The studies involving human participants were reviewed and approved by Washington University Institutional Review Board. Written informed consent to participate in this study was provided by the participants’ legal guardian/next of kin.

## Author Contributions

VM contributed to the conception and design of study, analyzed the data, and wrote the first draft of the manuscript. JB and CH contributed to the conception of the study and data collection. MR contributed to the data collection and database organization. HG contributed to statistical analysis. AK contributed to genetic data analysis and review. MC and MK contributed to the conception and design of the study, data collection, data analysis, and manuscript revision. All authors contributed to the article and approved the submitted version.

## Conflict of Interest

The authors declare that the research was conducted in the absence of any commercial or financial relationships that could be construed as a potential conflict of interest.

## Publisher’s Note

All claims expressed in this article are solely those of the authors and do not necessarily represent those of their affiliated organizations, or those of the publisher, the editors and the reviewers. Any product that may be evaluated in this article, or claim that may be made by its manufacturer, is not guaranteed or endorsed by the publisher.
